# NVP-BKM120, a novel PI3K inhibitor, shows synergism with a STAT3 inhibitor in human gastric cancer cells harboring KRAS mutations

**DOI:** 10.3892/ijo.2011.1290

**Published:** 2011-12-08

**Authors:** EUNJU PARK, JINAH PARK, SAE-WON HAN, SEOCK-AH IM, TAE-YOU KIM, DO-YOUN OH, YUNG-JUE BANG

**Affiliations:** 1 Cancer Research Institute, Seoul National University Hospital, Seoul National University College of Medicine, Seoul, Republic of Korea; 2 Department of Internal Medicine, Seoul National University Hospital, Seoul National University College of Medicine, Seoul, Republic of Korea; 3 Department of Molecular Medicine and Biopharmaceutical Sciences, Graduate School of Convergence Science and Technology, Seoul National University, Seoul, Republic of Korea

**Keywords:** BKM120, phosphoinositide 3-kinase, STAT3, KRAS, gastric cancer

## Abstract

Aberrations of Phosphoinositide 3-kinase (PI3K)/AKT signaling are frequently observed in many types of cancer, promoting its emergence as a promising target for cancer treatment. PI3K can become activated by various pathways, one of which includes RAS. RAS can not only directly activate the PI3K/AKT pathway via binding to p110 of PI3K, but also regulates mTOR via ERK or RSK independently of the PI3K/AKT pathway. Thus, actively mutated RAS can constitutively activate PI3K signaling. Additionally, in RAS tumorigenic transformation, signal transducer and activator of transcription 3 (STAT3) has been known also to be required. In this study, we examined the efficacy of NVP-BKM120, a pan-class I PI3K inhibitor in human gastric cancer cells and hypothesized that the combined inhibition of PI3K and STAT3 would be synergistic in KRAS mutant gastric cancer cells. NVP-BKM120 demonstrated anti-proliferative activity in 11 human gastric cancer cell lines by decreasing mTOR downstream signaling. But NVP-BKM120 treatment increased p-AKT by subsequent abrogation of feedback inhibition by stabilizing insulin receptor substrate-1. In KRAS mutant gastric cancer cells, either p-ERK or p-STAT3 was also increased upon treatment of NVP-BKM120. The synergistic efficacy study demonstrated that dual PI3K and STAT3 blockade showed a synergism in cells harboring mutated KRAS by inducing apoptosis. The synergistic effect was not seen in KRAS wild-type cells. Together, these findings suggest for the first time that the dual inhibition of PI3K and STAT3 signaling may be an effective therapeutic strategy for KRAS mutant gastric cancer patients.

## Introduction

Gastric cancer is the second most common cause of cancer-related death worldwide (1). Gastric adenocarcinoma has a poor outcome since high percentage of cases present with advanced disease. Chemotherapy has been considered to be useful treatment for advanced gastric cancer, but its current 5-year survival rate is less than 20% (1,2). Accordingly, the unmet need of effective treatment has led to an intensive effort to examine molecular regulators. Furthermore, based on the previous research that gastric cancer results from accumulated genetic alterations, which affect essential cellular functions for tumorigenesis, investigations to find a good predictive biomarker for targeted therapy have been undertaken in recent years in order to improve present therapeutics (1,3).

The PI3K/AKT pathway is known to play a key role in regulating various cellular processes, such as proliferation, growth, apoptosis, cytoskeletal rearrangement and cell metabolism (4,5). In gastric cancer, the PI3K/AKT signaling is inappropriately activated through mutation or alteration of many components of the PI3K pathway. Up to now, the mechanisms observed widely for PI3K/AKT activation in gastric cancer include somatic activating mutations and amplifications in p110α (6–8), loss of the PTEN tumor suppressor (8), and genetic amplifications of AKT1 (9). Preclinical study of human gastric cancer cell lines has demonstrated the anti-proliferative effect of PI3K inhibition by LY294002 or mTOR inhibition by everolimus and evidenced the synergistic efficacy with 5-fluorouracil or sunitinib, indicating a role for the PI3K/AKT pathway in gastric cancer carcinogenesis (10–12). In addition to gastric adenocarcinoma, the PI3K/AKT pathway has been an attractive target in clinical studies of various human cancers.

Agents targeting PI3K/AKT pathway in clinical development are pure PI3K inhibitors including NVP-BKM120, dual PI3K-mTOR inhibitors, AKT inhibitors and mTOR inhibitors. Isoform-specific PI3K inhibitors are also emerging.

According to previous studies, specific genetic alterations, such as HER2 amplification and PIK3CA mutation, were revealed as biomarkers for sensitivity to the PI3K inhibitor in breast cancer (13). However, cancers harboring KRAS mutations are likely to be insensitive to single-agent PI3K inhibitors and showed synergism in combination treatment with MEK inhibitors (14,15). In other words, KRAS mutant cancers insensitive to single treatment of PI3K inhibitors seem to induce at least one signaling mediator in the alternate pathway, which contributes to resistance. Thus, combined inhibition is required to suppress activation of other pathways and feedback loop-induced activation of other oncogenic signaling pathways, resulting in more potent induction of apoptosis.

The STAT pathway is another possible inducible pathway in response to PI3K inhibition and recently, STAT3 has been reported as an essential molecule in RAS oncogenic transformation (16). STATs are latent transcription factors that are involved in cell proliferation, survival, angiogenesis and immunosuppression (17). In diverse cancers including gastric cancer, the STAT pathway, especially STAT3, is constitutively activated and plays a major role in tumorigenesis (17,18). Thereby, an effort for directly or indirectly targeting the STAT signaling has been made to develop a new approach for effective cancer therapy. For example, preclinical studies of inhibition of STAT3 by STAT3 inhibitors or JAK2 inhibitors showed potent anti-tumor activity in cancers including solid tumors as well as myeloma (19,20).

In the present study, we characterized the antitumor effects exerted by Class I PI3K single inhibition and combination with STAT3 inhibition in gastric cancer cell lines for the first time. Results indicate that NVP-BKM120, a pan-class I PI3K inhibitor, is able to inhibit mTOR downstream activation, but induces the phosphorylation of AKT and the activation of p-ERK or p-STAT3 in KRAS mutant gastric cancer cells. The combination of NVP-BKM120 and AG490, a STAT3 inhibitor, showed a synergism leading to apoptosis, but this synergism was only observed in cells harboring mutant KRAS.

Thus, our result suggests that dual inhibition of PI3K and STAT3 signaling may be an effective therapeutic strategy for KRAS mutant gastric cancer patients.

## Materials and methods

### Cell lines

Human gastric cancer cell lines (SNU-1, -5, -16, -216, -484, -601, -620, -638, -668 and -719) were obtained from the Korean Cell Line Bank (21) and AGS was purchased from the American Type Culture Collection. All cell lines were maintained in RPMI-1640 supplemented with 10% fetal bovine serum (Hyclone Laboratories, Inc., Logan, UT, USA) and 10 μg/ml gentamicin (Cellgro, Herndon, VA, USA) at 37°C in a 5% CO_2_ humidified atmosphere.

### Reagents

**Figure 1:**
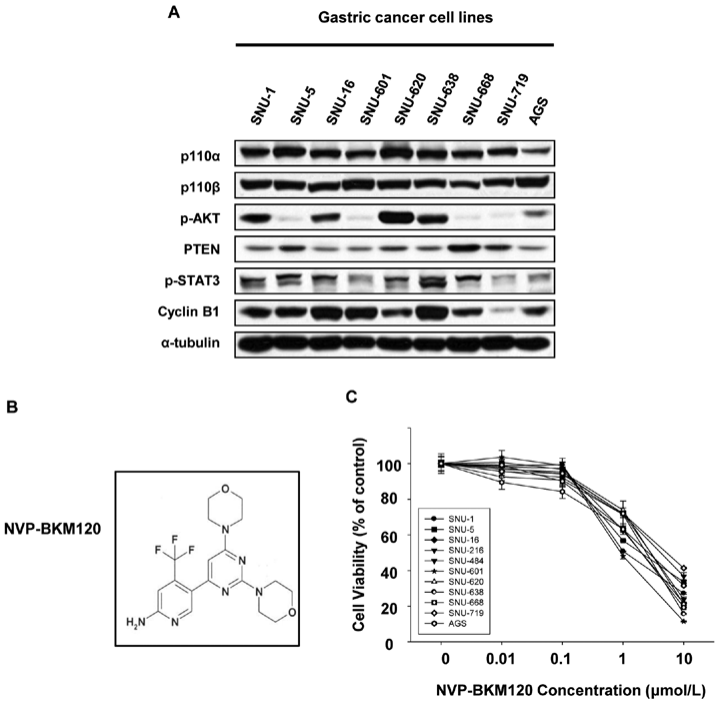
NVP-BKM120 effectively suppresses cell viability in human gastric cancer cells. (A) Panels of 11 human gastric cancer cell lines were incubated in medium containing 10% serum, then harvested 24 h after plating and probed with the indicated antibodies. (B) Chemical structures of NVP-BKM120, a pan-class I PI3K inhibitor. (C) Proliferation and biological activity were measured by tetrazolium dye assay. Data shown are means ± SD of four replicate wells. Representative experimental data from three independent experiments.

NVP-BKM120, a pan-class I PI3K inhibitor, was generously provided by Novartis Pharma AG (Basel, Switzerland) (Fig. 1B). NVP-BKM120 inhibits wild-type p110α (IC_50_ 35 nM), with high selectivity toward protein kinases and shows significant antitumor activity in animal models (in the Novartis brochure). It has been in Phase I clinical trials for solid tumors. AG490, a STAT3 inhibitor, was purchased from Sigma-Aldrich. Stock solutions for both drugs were prepared in dimethyl sulfoxide (DMSO) and stored at −20°C. NVP-BKM120 and AG490 were diluted in DMSO prior to each experiment, and the final concentration of DMSO was <0.1%.

### Antibodies and Western blotting

Cells were grown in 100 mm dishes and treated with NVP-BKM120 or AG490 for the indicated concentrations and time. Cells were washed twice with ice-cold phosphate-buffered saline and lysed in ice-cold lysis buffer (50 mM Tris-HCl, pH 7.5, 1% NP-40, 0.1% sodium deoxycholate, 150 mM NaCl, 50 mM NaF, 1 mM sodium pyrophosphate, 1 mM EDTA, and protease inhibitors). 20 μg of total proteins were resolved using sodium dodecyl sulfate-polyacrylamide gel electrophoresis. The resolved proteins were transferred to nitrocellulose membranes and probed with antibodies. Lysates were incubated with antibody overnight at 4°C. Detection was conducted using an enhanced chemiluminescence system (Amersham Pharmacia Biotech). Antibodies against p110α, p-AKT, p-p70S6K, p-4E-BP1, 4E-BP1, p-STAT3, p-ERK, and Cyclin D1 were purchased from Cell Signaling Technology. Anti-p110β antibody was acquired from Millipore. Anti-p27 [Kip1], anti-IRS-1 and anti-Cleaved PARP were acquired from BD Transduction Laboratories. Cyclin B1, PTEN and Actin antibodies were obtained from Santa Cruz Biotechnology. Anti-α-tubulin antibody was purchased from Sigma-Aldrich.

### Cell growth inhibition assay

Tetrazolium dye (MTT; Sigma-Aldrich) assays were used to evaluate the growth inhibitory effects of NVP-BKM120, AG490, or NVP-BKM120 plus AG490. The cells were seeded on 96-well plates at a density of 1,000–3,000 cells per well, incubated for 24 h, and then treated for 72 h with drugs at 37°C. After drug treatment, MTT solution was added to each well and incubated for 4 h at 37°C before the removal of the media. DMSO was then added and mixed thoroughly for 20 min at room temperature. Cell viability was determined by measuring absorbance at 540 nm in a microplate reader (VersaMax, Molecular Devices). The drug concentrations required to inhibit cell growth by 50% were determined through interpolation from the dose-response curves (CalcuSyn, Biosoft). Four replicate wells were used for each analysis, and at least three independent experiments were conducted. The data from replicate wells are presented as the mean numbers of remaining cells, with 95% confidence intervals.

### Cell cycle analysis

Cells were seeded in 60-mm dishes and treated with NVP-BKM120, AG490 or NVP-BKM120 plus AG490 for 72 h. Floating and adherent cells were collected by trypsinization and washed once with PBS. Cells were incubated in 70% ethanol at −20°C overnight, treated with 50 μg/ml RNase A, and stained with 50 μg/ml propidium iodide. The cell DNA content (10,000 cells per experimental group) was determined with a flow cytometer (FACS Canto™II, Becton-Dickinson Biosciences) equipped with a ModFit LT program (Verity Software House, Inc.). The experiments were repeated three times.

### Statistical analysis

An unpaired two-tailed t-test was used to determine the significance of change in the levels of cell viability and cell cycle analysis between the different treatment groups. Differences between groups were considered statistically significant at P<0.05.

## Results

### Anti-proliferative effects of NVP-BKM120 on human gastric cancer cell lines

The baseline levels of p110α, p110β, PTEN, phosphorylated AKT, ERK, and STAT3 in human gastric cancer cell lines were measured by Western blotting. The levels of PTEN, p-AKT, p-ERK and p-STAT3 were expressed to varying degrees in our panel of gastric cancer cell lines and p110α and p110β were expressed to similar degree in all cell lines (Fig. 1A).

To assess the anti-proliferative effects of a pan-class I PI3K inhibitor, NVP-BKM120, on human gastric cancer cells, we exposed 11 human gastric cancer cell lines with different modulations of PI3K/AKT cascade (Table I). Cell growth of all cell lines tested was effectively suppressed by NVP-BKM120 with IC_50_ values ranging from approximately 0.8 to 3 μmol/l (Table I, Fig. 1C). In our panel of gastric cancer cell lines, a correlation between the anti-proliferative effect of NVP-BKM120 and the genetic alterations including HER2 amplification, KRAS mutation, and both PIK3CA and KRAS mutation at the same time, was not found (Fig. 1A). Additionally, the baseline levels of Cyclin B1 was also irrelevant to the sensitivity to NVP-BKM120 differently from a previous study reporting that cells resistant to PI3K inhibitors expressed high levels of Cyclin B1 (15).

### The effect of NVP-BKM120 on PI3K/AKT/mTOR signaling in human gastric cancer cells

**Figure 2:**
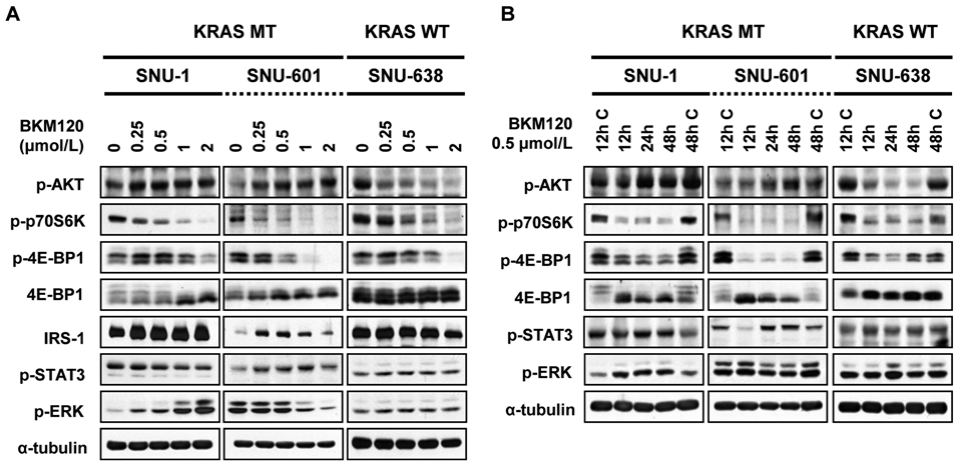
NVP-BKM120 inhibits mTOR signaling but induces STAT and/or ERK signaling. SNU-1, SNU-601 and SNU-638 were incubated in medium containing 10% serum for 24 h in the presence of various concentrations of NVP-BKM120 (A) or to 1 μmol/l NVP-BKM120 (B) from 12 to 48 h. Cells were then lysed and whole-cell lysates were immunoprecipitated with indicated antibodies. α-tubulin was used as a loading control. Representative experimental data from three independent experiments.

We next evaluated how inhibition of PI3K activity by NVP-BKM120 affects downstream AKT and mTOR activities. Cells were exposed to increased concentrations of NVP-BKM120 for 24 h and then subjected to Western blotting of phosphorylated AKT, 4E-BP1, p70S6K, ERK and STAT3. Dose- and time-response experiments revealed that in SNU-1, SNU-601, and SNU-638 cells, inhibition of PI3K activity resulted in an indirect downstream inhibition of AKT (p70S6K, 4E-BP1, through mTOR) (Fig. 2). However, unlike SNU-638 cells, SNU-1 and SNU-601 cells, which are KRAS mutants, showed increase of AKT phosphorylation in both dose- and time-dependent manner. Presumably, we assumed that it might be due to release of negative feedback loops of AKT signaling. The best-known mechanism includes insulin receptor substrate-1 (IRS-1) as an intermediary in the PI3K/AKT/mTOR negative feedback loop (22,23). Thus, we checked the dephosphorylation status of IRS-1 along with the increasing level of AKT. As shown in Fig. 2A, NVP-BKM120 induced IRS-1 total protein levels in SNU-1 and SNU-601 cell lines, which implies an increase in IRS-1 stability and association with PI3K, but not in SNU-638 cells.

Intriguingly, single treatment of NVP-BKM120 induced an alternate pathway in KRAS mutant gastric cancer cell lines in a cell line-specific manner. First, phosphorylation of ERK became induced in SNU-1 cells but decreased in SNU-601 cells though both cell lines are KRAS mutants. Second, p-STAT3 was increased in SNU-601 cells in a dose- and time-dependent manner, but slightly decreased in SNU-1 cells (Fig. 2). In contrast to these cell lines, SNU-638 cells did not show any induction of other pathways. This result coincides with previous research with PI3K inhibitors that PI3K inhibitor single treatment induces at least one signaling mediator in the alternate pathway (24).

### Combined inhibition of PI3K and STAT3 is synergistic in human gastric cancer cells harboring mutated KRAS

As Fig. 2 showed different activation of the other pro-survival pathways in KRAS mutants, we next studied the combination effect of NVP-BKM120 and other inhibitors. Considering the compensatory relationship between RAS/RAF/ERK and PI3K/AKT/mTOR pathways, KRAS mutant cancer cell lines have shown a synergistic effect of PI3K and MEK inhibitors. However, in our panel of gastric cancer cell lines while SNU-1 cells showed increase in phosphorylation of ERK, SNU-601 cells showed its decrease along with PI3K inhibition. Also, SNU-601 cells showed activation of STAT3. Since previous research demonstrated that STAT3 is required in KRAS-driven oncogenic transformation (16), we hypothesized that dual inhibition of PI3K and STAT3 would be effective in KRAS mutant gastric cancer cell lines. We used AG490 as a STAT3 inhibitor.

**Figure 3:**
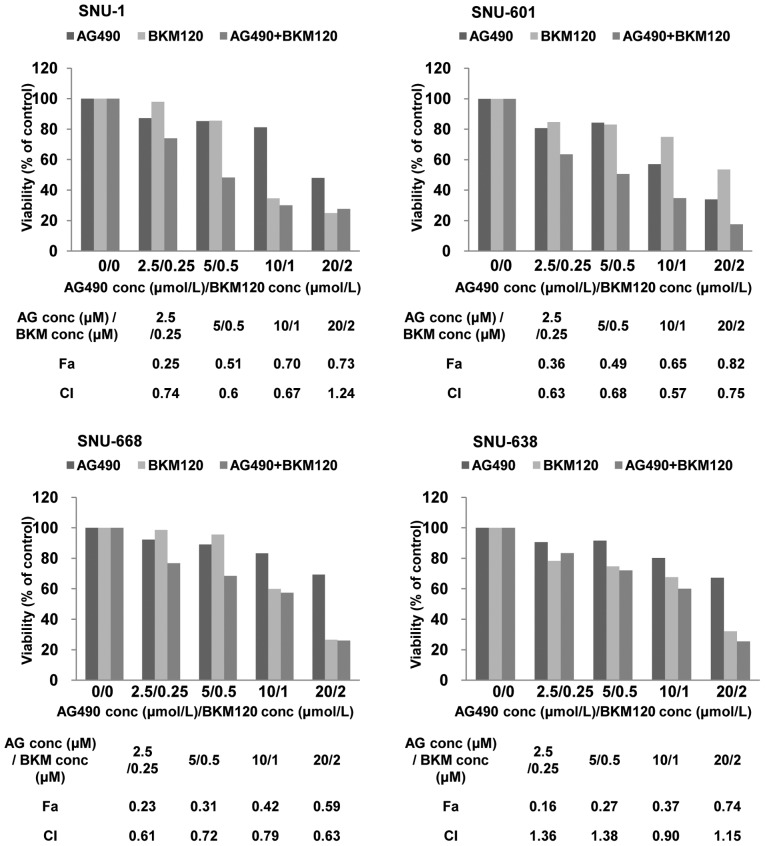
Combination of NVP-BKM120 and AG490 shows synergism in cells harboring mutated KRAS. The combination of NVP-BKM120 and AG490 was mixed in the molar ratio of 10:1 in SNU-1, SNU-601, SNU-668 and SNU-638 cells. Four cell lines were exposed to treatments for 72 h, and cell viability was determined. CI<1, synergistic effect; CI=1, additive effect; CI>1, antagonistic effect. Representative experimental data from three independent experiments.

To characterize the level of the interaction (synergistic, additive or antagonistic) between NVP-BKM120 and AG490, combination index (CI) values were calculated based on the Chou and Talalay median-effect principle (25). A CI is 1 for additive interactions, greater than 1 for antagonistic interactions, and less than 1 for synergistic interactions. As shown in Fig. 3, the combination of NVP-BKM120 and AG490 induced synergistic killing of KRAS mutant gastric cancer cells at different dose combinations and the synergistic effect was especially distinctive at low dose combinations, contrast to KRAS wild-type SNU-638 cells showing antagonistic effect at low dose combinations. In aggregate, we found that PI3K inhibition by NVP-BKM120 cooperated with AG490 in gastric cancer cells harboring mutated KRAS.

### The combination of NVP-BKM120 and AG490 induces apoptosis

**Figure 4:**
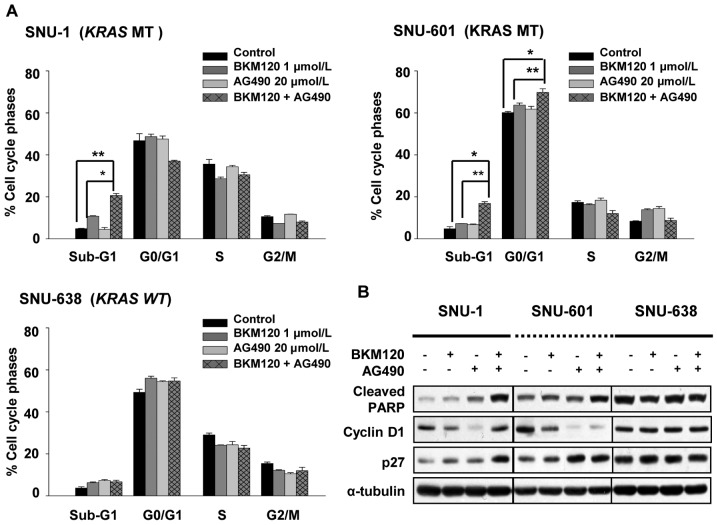
Combination of NVP-BKM120 and AG490 inhibits progression of SNU-1 and SNU-601 cells through apoptosis. (A) Cells were incubated in medium containing 10% serum for 72 h in the presence of NVP-BKM120, AG490, or combination of NVP-BKM120 and AG490 in indicated concentrations, after which they were fixed, stained with propidium iodide, and analyzed for cell cycle distribution by flow cytometry. (B) Cells were incubated in medium containing 10% serum for 72 h in the presence of NVP-BKM120, AG490 alone or combination of NVP-BKM120 and AG490. Cells were then lysed and whole-cell lysates were immunoprecipitated with indicated antibodies. α-tubulin was used as a loading control. Representative experimental data from three independent experiments; bars, ± SE. ^*^P<0.05; ^**^P<0.005.

To confirm the synergistic interaction of NVP-BKM120 with AG490, we evaluated the cell cycle distribution in SNU-1, SNU-601 and SNU-638 cells. We incubated SNU-1, SNU-601, and SNU-638 cells for 72 h in the presence of NVP-BKM120 alone, AG490 alone, or combination of NVP-BKM120 and AG490 in concentrations as indicated in Fig. 4A and did cell cycle analysis using flow cytometry. We found that concurrent treatment of NVP-BKM120 and AG490 leads to cell death in both SNU-1 and SNU-601 cells, and in SNU-601 cells growth arrest in the G1 phase of the cell cycle was detected as well.

We further investigated the relative expression levels of cell-cycle related proteins by Western blotting (Fig. 4B). The combination of NVP-BKM120 and AG490 induced expressions of cleaved PARP and p27 and down-regulated Cyclin D1 in SNU-601 cells.

### The effect of combined inhibition of PI3K and STAT3 on the signaling of human gastric cancer cells with mutated KRAS

**Figure 5:**
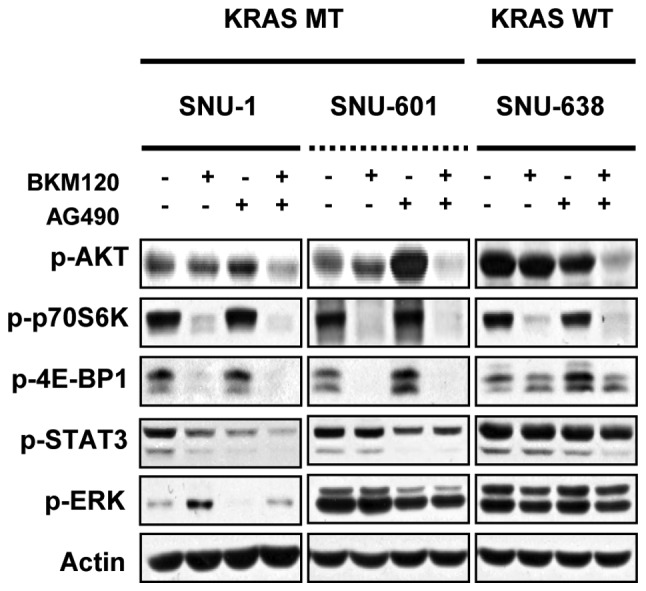
Combination of NVP-BKM120 and AG490 on the signaling of SNU-1, SNU-601 and SNU-638. Cells were incubated in medium containing 10% serum for 48 h in the presence of 1 μmol/l NVP-BKM120, 20 μmol/l AG490 alone or combination of NVP-BKM120 and AG490. Cells were then lysed and whole-cell lysates were immunoprecipitated with antibodies indicated. Actin was used as a loading control. Representative experimental data from three independent experiments.

Because we could show that combined treatment of NVP-BKM120 and AG490 caused synergistic inhibition of proliferation and induction of apoptosis in KRAS mutant gastric cancer cell lines, SNU-1 and SNU-601, we next examined their effects alone and in combination on signaling pathways. As shown in Fig. 5, combined inhibition of PI3K and STAT3 inhibited the phosphorylation of AKT and p70S6K in SNU-1, SNU-601 and SNU-638 cells. The phosphorylation of 4E-BP1 and STAT3 were decreased only in SNU-1 and SNU-601 cells. In KRAS wild-type SNU-638 cells phosphorylation of STAT3 was slightly decreased and no significant change in expression levels of p-ERK and p-4E-BP1 was detected.

## Discussion

The PI3K/AKT signaling axis is generally deregulated by various genetic changes in solid tumors. The aberrant activation of the PI3K/AKT pathway contributes to cell survival, protein synthesis and cell metabolism. In gastric cancer, genetic mutation/amplification of PIK3CA, AKT1 and KRAS, and loss of heterozygosity of PTEN have been recognized so far (8,9). However, it is not well understood how these changes qualitatively or quantitatively affect PI3K signaling and whether gastric cancer harboring these mutations is addicted to PI3K signaling and will be sensitive to PI3K inhibitors.

In this study, we found that during PI3K inhibition, AKT, ERK and/or STAT3 are activated as shown by the increased levels of phosphorylation in gastric cancer cells. This is further supported by previous studies on PI3K pathway that PI3K inhibitor single treatment appears to be not sufficient as it induces at least one signaling mediator in the alternate pathway. One possible reason for the limited efficacy of single PI3K inhibition is the presence of feedback loops. First, as shown in Fig. 2A, in SNU-1 and SNU-601 cells along with PI3K inhibition by NVP-BKM120 single treatment, AKT is activated through activated mTOR/S6K/IRS-1 negative feedback mechanism. This is in accordance with recent studies that mTOR or AKT inhibition increases PI3K activity and enhances AKT-independent PI3K pathway (23,26,27). Second, our data showed ERK or STAT3 activation after PI3K inhibition alone in KRAS mutant cancer cell lines as a previous study showed that inhibition of mTORC1 induces RAS pathway as well (28). This compensatory activation of other pro-survival pathways due to inhibition of the PI3K pathway has been reported as prominent between the PI3K/AKT and RAS/MAPK pathways. The PI3K/AKT and RAS/MAPK signaling pathways influence each other rather than function independently, resulting in active and complex crosstalk. For instance, it has been reported that the interaction of RAS with p110α is required for RAS-driven oncogenic transformation (29). In this respect, in cancers, RAS activation may limit the activity of single-agent PI3K inhibitors. One of the most typical resistant mechanisms to PI3K inhibition is activating mutations in the RAS/MAPK pathway (14,15,24,30,31). Therefore, to combine inhibition of the PI3K/AKT pathway with inhibition of the RAS/MAPK pathway is a possible approach in order to overcome a compensatory interaction between these pathways (14,24,32).

In contrast to the relation between PI3K and RAS pathways, there has been only a few reports on interaction between PI3K and STAT pathways. STAT3 is a latent cytoplasmic transcription factor and its pathway is one of the key signaling pathways of which deregulation drives tumorigenesis. STAT3 transmits signals to the nucleus where STAT3 binds to specific DNA promoter and regulates gene expression (17,33). In many human cancers, STAT3 is constitutively activated and has been described as a novel molecular target for cancer drug discovery.

**Figure 6:**
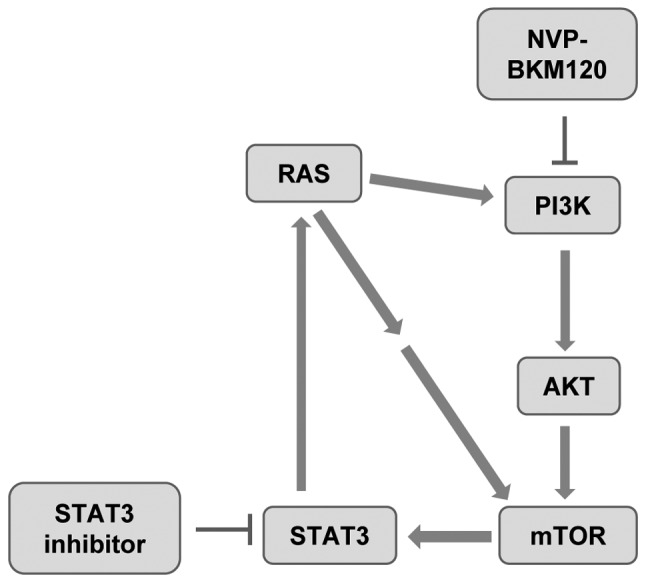
Schematic representation of RAS/PI3K/mTOR/STAT3 signaling. Constitutively active RAS can regulate the STAT3 pathway in dependent of the canonical PI3K/AKT pathway. In addition, RAS can directly activate mTOR via RSK-mediated phosphorylation of Raptor. mTOR as a kinase fully activates STAT3, which is essential for RAS oncogenic transformation. In RAS mutant cancer cells, therefore, despite suppression of PI3K by NVP-BKM120, the RAS signaling still enables to maintain oncogenic transformation through activation of the mTOR/STAT3 signaling. Combination of NVP-BKM120 and AG490 may result in a synergistic effect on gastric cancer harboring KRAS mutation.

According to previous studies, mTOR as a serine kinase positively activates STAT3 (34,35). For RAS-dependent malignant transformation, activated STAT3 is essential (16). In addition, activated RAS/MAPK signaling can directly regulate mTOR via p90 ribosomal S6 kinase (RSK)-mediated phosphorylation of Raptor independently of the PI3K/AKT pathway (36). Taken together, although a PI3K inhibitor suppresses the PI3K/AKT/mTOR signaling pathway, oncogenic RAS activates RAS/mTOR/STAT3 signaling (Fig. 6).

Therefore, we hypothesized that in KRAS mutant gastric cancer cells, the blockade of both PI3K/AKT/mTOR and KRAS/mTOR/STAT pathways using NVP-BKM120 and AG490 would be synergistic. We used median-effect analysis of dose-response curves to calculate the CI values and the results were represented as synergy in KRAS-mutated gastric cancer cell lines, SNU-1, SNU-601 and SNU-668. The synergistic effect was demonstrated by induction of apoptosis and by changes of signaling molecules along with combination treatment of NVP-BKM120 and AG490 in SNU-1 and SNU-601 cells.

In case of SNU-668 cells, the effect of concurrent PI3K and STAT3 inhibition on the expression of signaling molecules might be stronger than SNU-1 and SNU-601 cells. Based on the recent study a small reduction in PTEN gene expression can trigger cancer susceptibility, the higher level of PTEN in SNU-668 would play an enhanced role as a negative regulator of STAT3 and mTOR, consequently resulting in more significant molecular change of signaling (37). This is further supported by our previous result that the SNU-668 cell line is less sensitive to dual PI3K/mTOR inhibitor NVP-BEZ235, compared to other gastric cancer cell lines (data not shown).

To our knowledge, this is the first study to show that concurrent inhibition of PI3K and STAT signaling pathways is synergistically effective in KRAS mutant gastric cancer cells. Therefore, our study suggests the importance to select appropriate patient subpopulations for clinical study. PI3K blockade in combination with STAT3 inhibitors may benefit patients with gastric cancer exhibiting oncogenic KRAS.

## Figures and Tables

**Table I:** Sensitivity of NVP-BKM120 in human gastric cancer cell lines.

Cell lines	KRAS	Others	BKM120 IC_50_ (μmol/l)
SNU-601	MT, G12D		0.816±0.063
SNU-1	MT, G12D		1.082±0.028
SNU-668	MT, Q61K		1.579±0.074
AGS	MT, G12D	PIK3CA, E453K^a^	1.741±0.117
SNU-216	WT	HER2 amplification	2.692±0.082
SNU-5	WT	MET amplification	1.351±0.091
SNU-638	WT	MET amplification	2.282±0.053
SNU-16	WT	FGFR2 amplification	1.573±0.001
SNU-484	WT		1.728±0.045
SNU-620	WT		2.939±0.001
SNU-719	WT		3.037±0.032

Shown are IC_50_ values of each drug using tetrazolium dye (MTT) assays as described in Materials and methods.

a PIK3CA mutation status refer to the Cancer Genome Project database from the Wellcome Trust Sanger Institute (www.sanger.ac.uk).

MT, mutant; WT, wild-type.
